# Preferential Solvation of Mg^2+^ in Ethanol–Water Mixtures Probed by Intermolecular Coulombic Decay and Molecular Dynamics Simulations

**DOI:** 10.1021/acsphyschemau.6c00039

**Published:** 2026-08-06

**Authors:** Ouassim Hocine Hafiani, Alexandre M. Pereira, Luciano T. Costa, Michele Pugini, Shirin Gholami, Nicolas Velasquez, Florian Trinter, Iyas Ismail, Ricardo Marinho, Alexandra Mocellin, Arnaldo Naves de Brito, Carl Caleman, Lukáš Tomaník, Bernd Winter, Olle Björneholm, Gunnar Öhrwall

**Affiliations:** † Department of Physics and Astronomy, 8097 Uppsala University, Uppsala 75120, Sweden; ‡ MolMod-CS, Institute of Chemistry, 28110 Fluminense Federal University, Niterói 24020-141, Brazil; § 28259 Fritz-Haber-Institut der Max-Planck-Gesellschaft, Berlin 14195, Germany; ∥ 27051 Sorbonne Université, CNRS, Laboratoire de Chimie Physique-Matière et Rayonnement, Paris 75005, France; ⊥ Institute of Physics, 28127 Brasilia University, Brasília 70919-970, Brazil; # Institute of Physics, Federal University of Bahia, Salvador 40170-115, Brazil; ∇ Department of Applied Physics, Institute of Physics Gleb Wataghin, 344101 Campinas University, Campinas 13083-859, Brazil; ○ Department of Analytical Chemistry, 52735 University of Chemistry and Technology, Prague 16628, Czech Republic; ◆ MAX IV Laboratory, Lund University, Lund 22100, Sweden

**Keywords:** intermolecular Coulombic decay, solvation, preferential solvation, metal ions, molecular dynamics, liquid jet, electron spectroscopy

## Abstract

The microsolvation of Mg^2+^ ions in ethanol–water mixtures was investigated using intermolecular Coulombic decay (ICD) after Mg 1s ionization, a nonlocal relaxation process involving the ejection of valence electrons from molecules in the first solvation shell. As the solvent composition shifts from pure water to pure ethanol, the ICD spectral shape evolves, and its total intensity decreases by 20% relative to the local Mg KLL Auger decays. Spectral analysis reveals that Mg^2+^ ions are preferentially solvated by water; a substantial contribution from ethanol is observed only at high ethanol concentrations. Molecular dynamics simulations corroborate the experimental results, and together they provide quantitative understanding for the changes in solvation-shell composition, revealing a substantial enrichment of water in the solvation shell relative to the bulk solvent composition.

## Graphical abstract

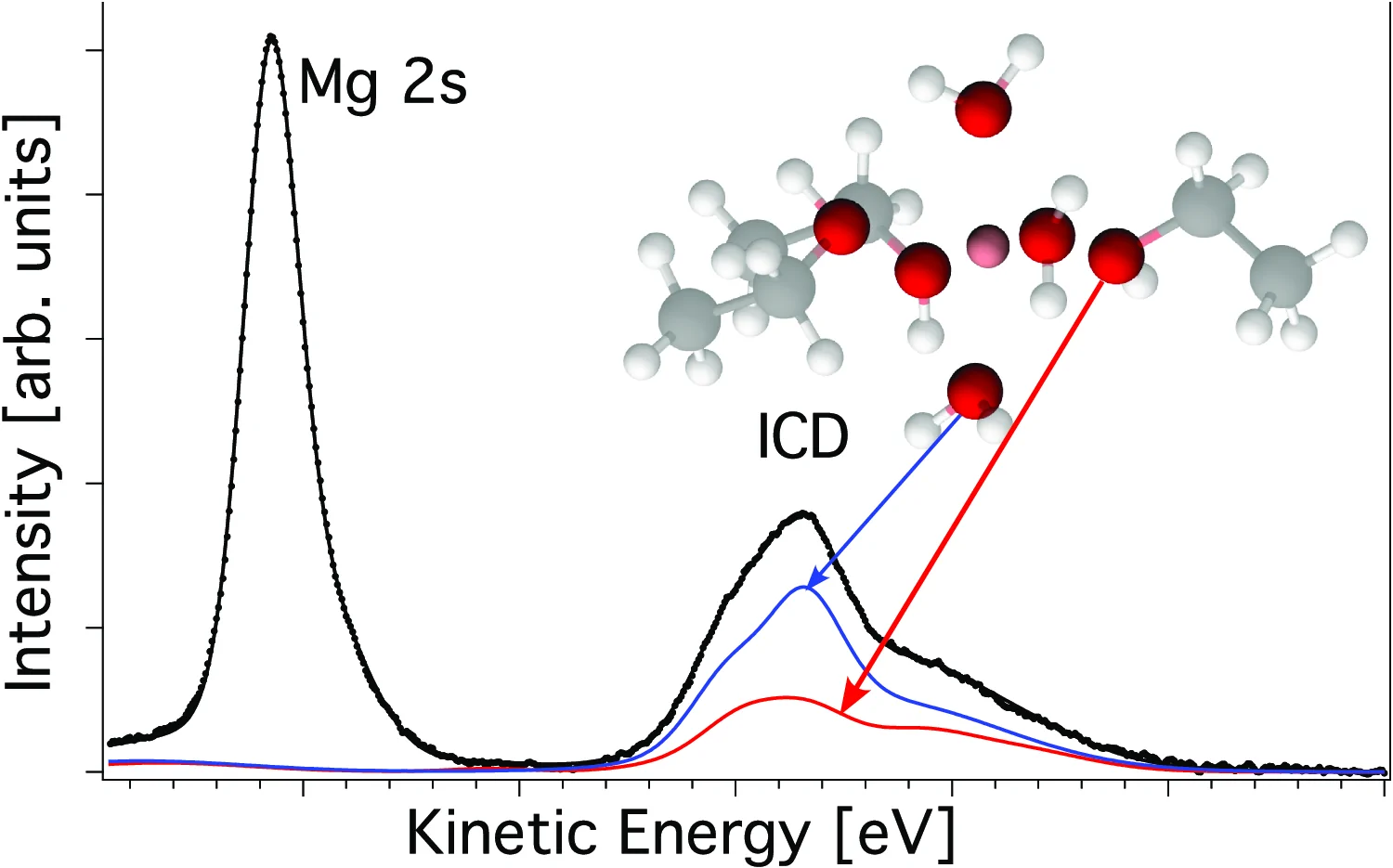

## Introduction

Complex environments consisting of more than one solvent and solute are important in a wide variety of cases, e.g., in batteries, in nature, and in living cells. In such environments, the solvation shell around a solute may not have the same composition as the bulk solution. For example, for a solute in a mixed solvent system, one solvent species may be enriched, and the other depleted in the solvation shell. This phenomenon is known as preferential solvation,^1,2^ and affects the solubility and stability of the solutes, influences reaction rates and chemical equilibria in solution. It is also important in biochemistry, for example, protein folding in water–cosolvent systems.^3–5^ Therefore, a molecular-level insight into the solvation of ions in such complex solutions is crucial to advance our understanding of numerous chemical and biological processes. However, probing solvation shells experimentally at the molecular scale remains a challenge.

Intermolecular Coulombic Decay (ICD) offers a powerful avenue for studying the local solvation shell, as it is sensitive only to the immediate molecular environment.^6–8^ For a metal ion in aqueous solution, M_*aq*_^*q*^, 1s core ionization leads to the M_*aq*_^*q*+1^ [1s]^−1^ core-hole state. For light elements, this will mainly decay via local KL_2,3_L_2,3_ Auger decay, M_*aq*_^*q*+1^ [1s]^−1^ → M_*aq*_^*q*+2^ [2p]^−2^ + e_Auger_^–^. In ICD the core-hole decay involves the ejection of an electron from the valence band of a neighboring water molecule W, M_*aq*_^*q*+1^ [1s]^−1^ + W → M_*aq*_^*q*+1^ [2p]^−1^ + W^+^[val]^−1^ + e_ICD_^–^. ICD has been observed for metal ions such as Mg^2+^, Al^3+^, K^+^, and Ca^2+^ in aqueous solutions.^9–13^ Moreover, ICD has been successfully applied to investigate various solvated systems, including the hydroxide-water network,^14^ and the solvation shell around metal ions, including contact ion pairs.^15,16^ Beyond the inorganic ions, ICD has been used to obtain quantitative information about the local coordination for the biologically relevant complex Mg^2+^–ATP.^17^ As a further step toward studying more complex environments such as those of biological relevance, we here investigate the solvation of a metal ion, Mg^2+^, in a mixture of two fully miscible polar solvents: water and ethanol. Magnesium, the 11th most abundant element in the human body by mass, was selected for its known, appreciable ICD signal following the ionization of the 1s core level in aqueous solution,^11^ and the high solubility of MgCl_2_ in both solvents. The solvation structure of Mg^2+^ in aqueous solution has been studied with experimental^18^ and theoretical^19^ approaches, indicating a 6-fold coordination of the ion. Constrained MD simulations of MgCl_2_ solvation in mixed water–ethanol solutions have been reported,^20^ but we are unaware of any experimental studies of solvation in such systems.

The immediate surrounding of an ion can be probed by methods such as EXAFS, which is sensitive to the local geometric structure around a specific center. In the case studied here, Mg^2+^ in solutions of water and ethanol, the experiment would be challenging, and we know of very few EXAFS studies of Mg in aqueous solution.^18^ Experimentally, the needed photon-energy range is not widely available, and the vacuum requirements of soft X-ray absorption measurements make experiments with volatile liquid solutions difficult to perform. Additionally, our molecular dynamics simulations (see below) show similar Mg–O distances and coordination numbers for all mixtures that we studied, making it uncertain whether EXAFS measurements would be sufficiently sensitive to changes in the solvation shell.

In this paper, we present a study of how the local solvation environment of Mg^2+^ in water–ethanol mixtures changes as a function of solvent mixing ratio. By combining experimental results and MD simulations, we obtain a comprehensive understanding of the solvation shell around Mg^2+^ in terms of composition, coordination number, and distances.

## Results and Discussion

We first present and analyze the experimental results, after which the results of the MD simulations will be presented, followed by a unifying discussion.

Figure 1 displays electron spectra from 0.5 M MgCl_2_ in pure water (mole fraction of ethanol χ_et_ = 0) and nearly pure ethanol (χ_et_ = 0.99). Similar spectra for intermediate mole fractions were also recorded, but are not shown. The spectra cover an electron kinetic-energy range that includes the Mg KL_2,3_L_2,3_ Auger features (around 1170–1180 eV, resulting from Mg^2+^ + hν → Mg^3+^ [1s]^−1^ → Mg^4+^ [2p]^−2^ + e_Auger_^–^), the KL_2,3_ ICD features (most intense around 1230–1245 eV, resulting from Mg^2+^ + W/E + hν → Mg^3+^ [1s]^−1^ + W/E → Mg^3+^ [2p]^−1^ + W/E^+^[val]^−1^ + e_ICD_^–^, where W/E denotes either a water or an ethanol molecule), and the Mg 2s photoelectron line (at ∼1224 eV).

**Figure 1:**
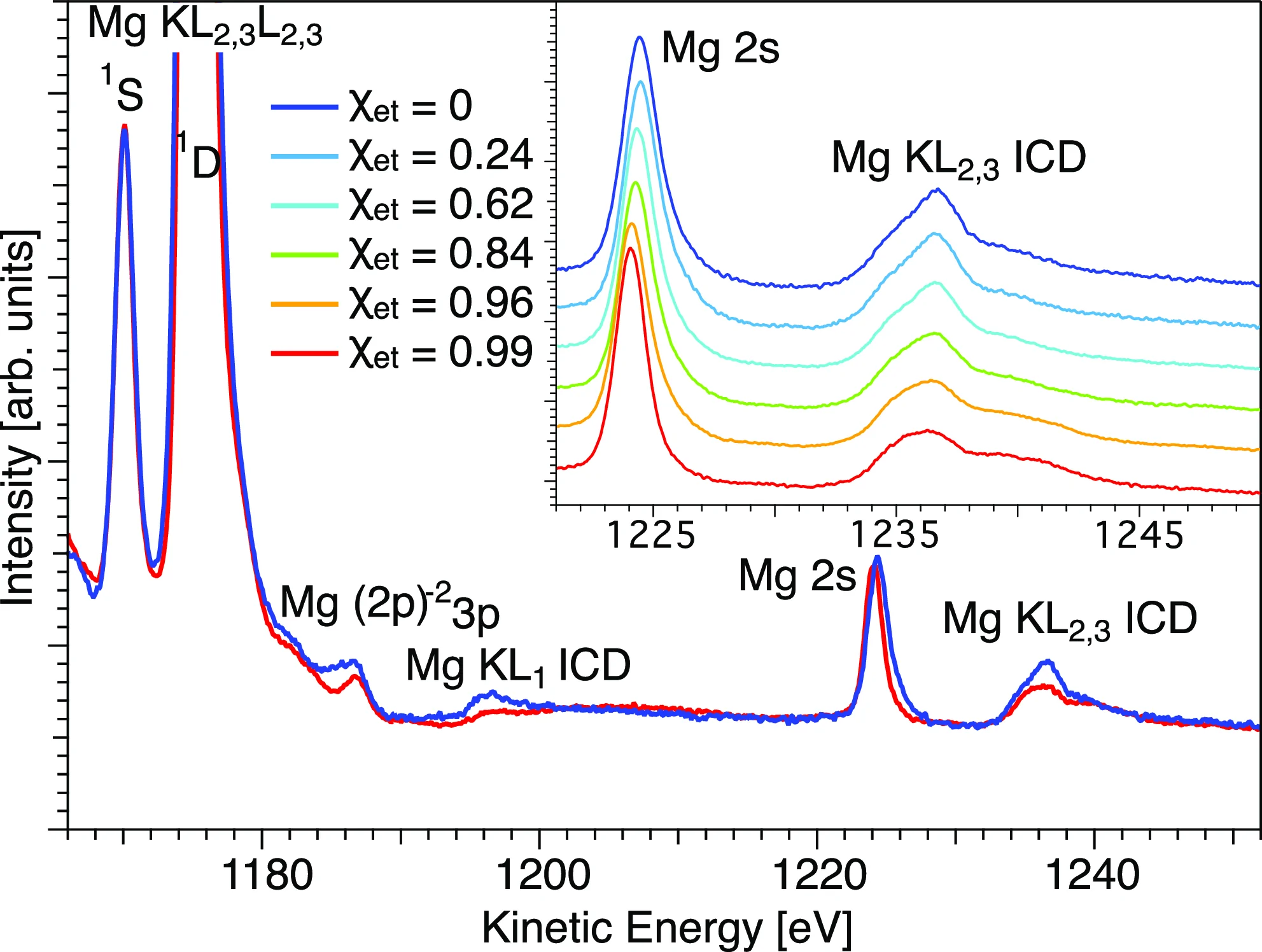
Overview spectra of the kinetic-energy region 1166 to 1253 eV for 0.5 M MgCl_2_ in ethanol (red) and water (blue). The region includes the Mg KL_2,3_L_2,3_ Auger features as well as Mg KL_2,3_ ICD features. At the chosen photon energy (1318.7 eV), the Mg 2s photoelectron feature appears close to the KL_2,3_ ICD feature. The spectra have been normalized to the Mg KL_2,3_L_2,3_^1^S peak area. Constant backgrounds have been subtracted to overlay the spectra in the region of the KL_2,3_ ICD feature for illustrative purposes. The inset shows electron spectra of the Mg 2s and KL_2,3_ ICD features for ethanol–water mixtures with varying molar fraction of ethanol (χ_et_), recorded at the same photon energy but with finer energy steps and improved statistics. The spectra are normalized to the Mg KL_2,3_L_2,3_^1^S feature and vertically offset for clarity.

The kinetic energy of the Mg KL_2,3_L_2,3_ features in aqueous solution has previously been reported,^11,21^ as well as the binding energy of Mg 2s.^10^ From the literature values, we have estimated the photon energy to be 1318.7 eV for the aqueous solution. The kinetic-energy scale in Figure 1 has been shifted so that the Mg KL_2,3_L_2,3_ features of the aqueous solution (χ_et_ = 0) align with the published values.^11,21^ The kinetic-energy scale for the other solutions was adjusted so that the Mg KL_2,3_L_2,3_^1^S feature aligned to the aqueous case. The spectra were also normalized so the Mg KL_2,3_L_2,3_^1^S peak has the same area for all solutions, based on the assumption that the KL_2,3_L_2,3_ decays involve relatively deep core levels and that the decay rate will therefore not be affected by the solvation.

In addition to the Mg KL_2,3_L_2,3_, KL_2,3_ ICD, and 2s features that will be most important for the following discussion, features from KL_1_ ICD, involving a Mg 2s electron in the decay, and from a 2p^–2^3p satellite state are observed. The KL_1_ ICD has previously been observed for aqueous solutions,^11^ as has the 2p^–2^3p state.^21^ The energy separation of the Mg KL_2,3_L_2,3_^1^S peaks and Mg 2s peaks decreases with ethanol concentration, indicating a systematic effect. However, since the photon energy was not sufficiently well calibrated throughout the experiment, except for the case of pure water, this cannot be stated with certainty.

It is immediately obvious from the spectra that, relative to the Mg KL_2,3_L_2,3_^1^S, the KL_2,3_ ICD features decrease in intensity for ethanol solvation compared to water. The most obvious explanation for this observation is that the probability of nonlocal ICD decreases as ethanol molecules solvate Mg^2+^ ions, since this will depend on, e.g., coordination number, ion–solvent-molecule distances, and orientation. Arguably, varying inelastic mean free paths (IMFPs) in the mixed systems could contribute, but this can be ruled out as explained in the Supporting Information.

Another observation is that the shape of the KL_2,3_ ICD feature changes, with the peak at 1236 eV becoming more prominent and sharper for water. In the inset of Figure 1, spectra are shown for ethanol–water mixtures with varying molar fraction of ethanol (χ_et_), covering a smaller region around the Mg 2s and KL_2,3_ ICD features, but recorded with a finer step size and improved statistics compared to the overview spectra. In the inset figure, a gradual change in the shape of the KL_2,3_ ICD feature with ethanol concentration can be seen. At first glance, the spectral shape for solutions with lower ethanol concentrations (χ_et_ = 0.24, 0.62) appears quite similar to that of pure water (χ_et_ = 0), and only at the highest ethanol concentrations (χ_et_ ≥ 0.84) does the shape change more substantially. The most immediate explanation for this observation is that water dominates the solvation shell at lower ethanol contents, even though the molar fraction of ethanol is relatively large, and that only at the highest ethanol concentrations does the contribution from ethanol solvation become dominant.

ICD involves electrons from the orbitals of the solvating molecules, and the final states of the KL_2,3_ ICD features are thus of the type Mg^3+^ [2p]^−1^ + W/E^+^[val]^−1^. The valence orbitals of water and ethanol have comparable energies,^23,24^ and we expect ethanol molecules to be oriented with the polar hydroxyl group toward the Mg^2+^ ion, as is borne out of the MD simulations. This causes the final states with W^+^[val]^−1^ and E^+^[val]^−1^ to overlap energetically, but the ICD signals are sufficiently different to allow the two contributions to be separated. However, a detailed understanding of the electronic decay spectra would require state-of-the-art *ab initio* calculations that are beyond the scope of this paper.

The solutions also contain the counterion Cl^–^, and if present in the solvation shell around the Mg^2+^ ion, Cl^–^ could potentially contribute to the ICD signal, as observed for Ca^2+^ at high concentrations.^16^ For the systems investigated here, we do not detect any such ICD signal; see SI for further details. We take this observation as evidence that Mg^2+^–Cl^–^ contact ion pairs are rare in both water and ethanol for a concentration of 0.5 M. The finding that contact ion pairs are scarce also provides a constraint on the MD simulations, as will be discussed below. Solvent-shared ion pairs, which have been suggested in aqueous solutions of MgCl_2_,^25^ would not be expected to contribute to the ICD signal because of the strong *R* ^–6^ distance dependence of the decay probability. We therefore assume only water- and ethanol-valence-band contributions to the ICD signal in the following analysis.

To quantify the composition of the solvation shell, we fitted the spectra according to a model described in detail in the Experimental section of the SI, and in Figure 2A example fits for χ_et_ = 0, χ_et_ = 0.84, and χ_et_ = 0.99 are shown. In this model, we assumed that the ICD spectral features remain unchanged when going from a pure solvent (water and ethanol) to a mixed solvent. Even though the solvation environment will influence the energies of the ICD final states to some degree, the transitions primarily involve localized valence orbitals that retain their chemical character. Furthermore, any subtle solvent-induced energetic shifts are negligible compared to the inherently large width of the ICD features. Consequently, we find that this model provides convincing fits for the full concentration range and serves as a robust approximation for the analysis.

**Figure 2:**
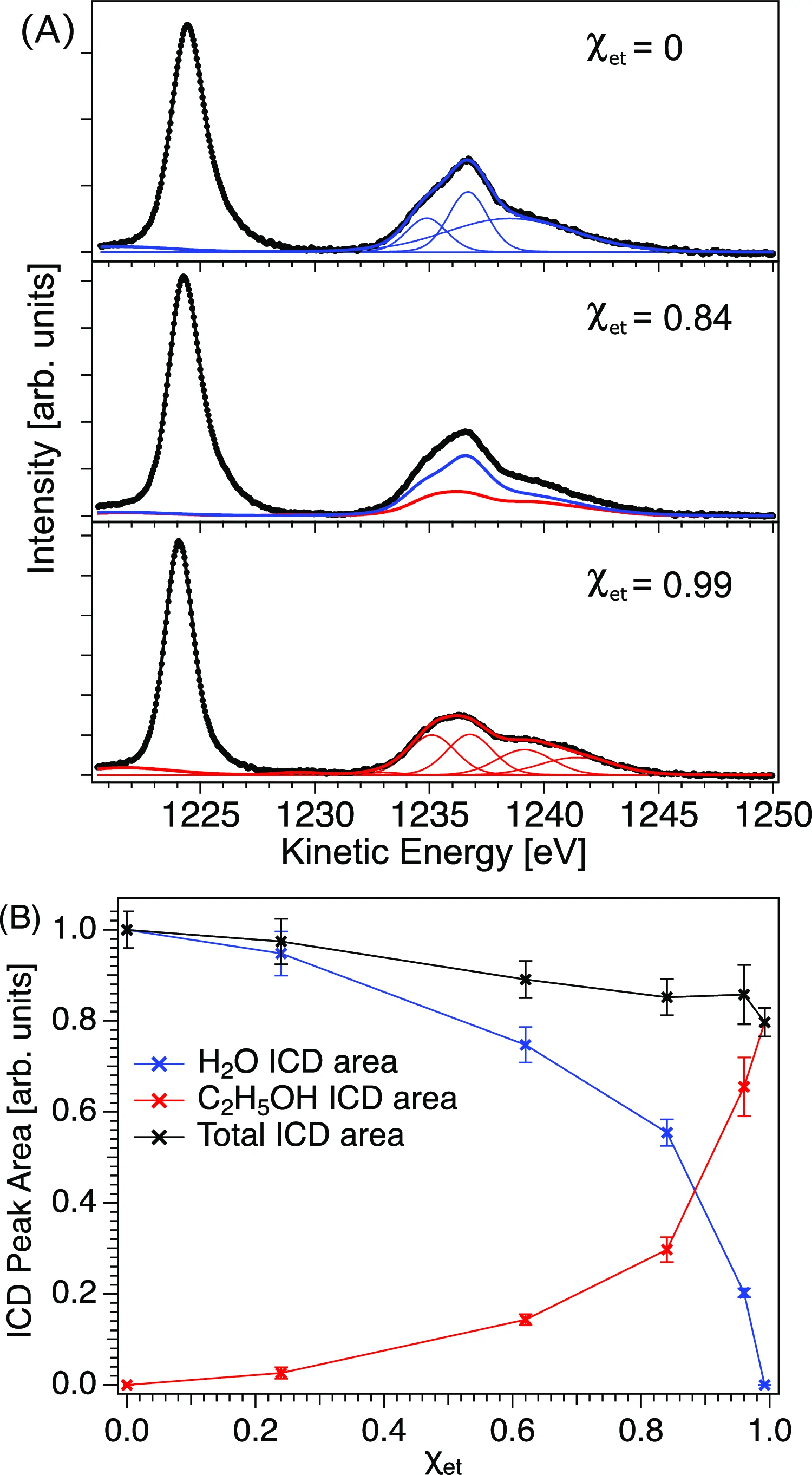
(A) Examples of fits of the ICD features for pure water (χ_et_ = 0, top), an ethanol–water mixture (χ_et_ = 0.84, center), and nearly pure ethanol (χ_et_ = 0.99, bottom). Filled black circles—experimental data, thin blue (red) lines—individual ICD fitting peaks for water (ethanol), thick blue (red) line—total ICD shape for water (ethanol), thick black line—total fit. (B) Fitted ICD peak areas as a function of χ_et_, showing the water contribution (blue crosses), the ethanol contribution (red crosses), and the total ICD signal (black crosses).

In Figure 2B, the total peak areas (normalized to the Mg KL_2,3_L_2,3_^1^S feature) obtained from fitting the ICD spectra with one water contribution and one ethanol contribution are shown as a function of ethanol concentration. The water and ethanol ICD features overlap substantially, but the least-squares fitting procedure shows that, while the water signal contribution in the mixed solutions decreases with decreasing water content, it remains considerably larger than expected from the molar fraction alone. The intensity of the ICD features is proportional to the decay rate and depends on the coordination number, ion–molecule distance, and orientation.^6,7^ The larger spectral contribution from water therefore does not, by itself, imply that the solvation shell around the Mg^2+^ ions contains an overrepresentation of water. However, the fact that the total ICD intensity decreases by only about 20% when going from pure water to pure ethanol shows that the probability of ethanol-mediated ICD is not dramatically lower than that of water-mediated ICD and points to a relatively similar local environment around the Mg^2+^ ion for the two cases. To summarize the experimental results obtained so far, we conclude that the ICD spectra indicate a substantial preference for water over ethanol in the solvation of the Mg^2+^ ions, while no signature of Mg^2+^-Cl^–^ contact ion pairing is observed.

A measure that we believe better describes the coordination around the ion can be obtained from the peak areas by scaling the ethanol contributions by a factor that equalizes the total intensity for pure ethanol to that of pure water (1/0.797, see Table S1), to compensate for the lower decay rate for ethanol coordination. The water and scaled ethanol contribution peak areas can then be assumed to correspond to the relative preponderances of the molecules around the ion. The sum of the water and the scaled ethanol peak areas varies within ±5%, giving an indication of the reliability of this approach. In Figure 3A, the branching ratio of the water signal and the scaled ethanol signal, which should be a measure of the relative coordination around the Mg^2+^ ion, is plotted as a function of the mole fraction of ethanol.

**Figure 3:**
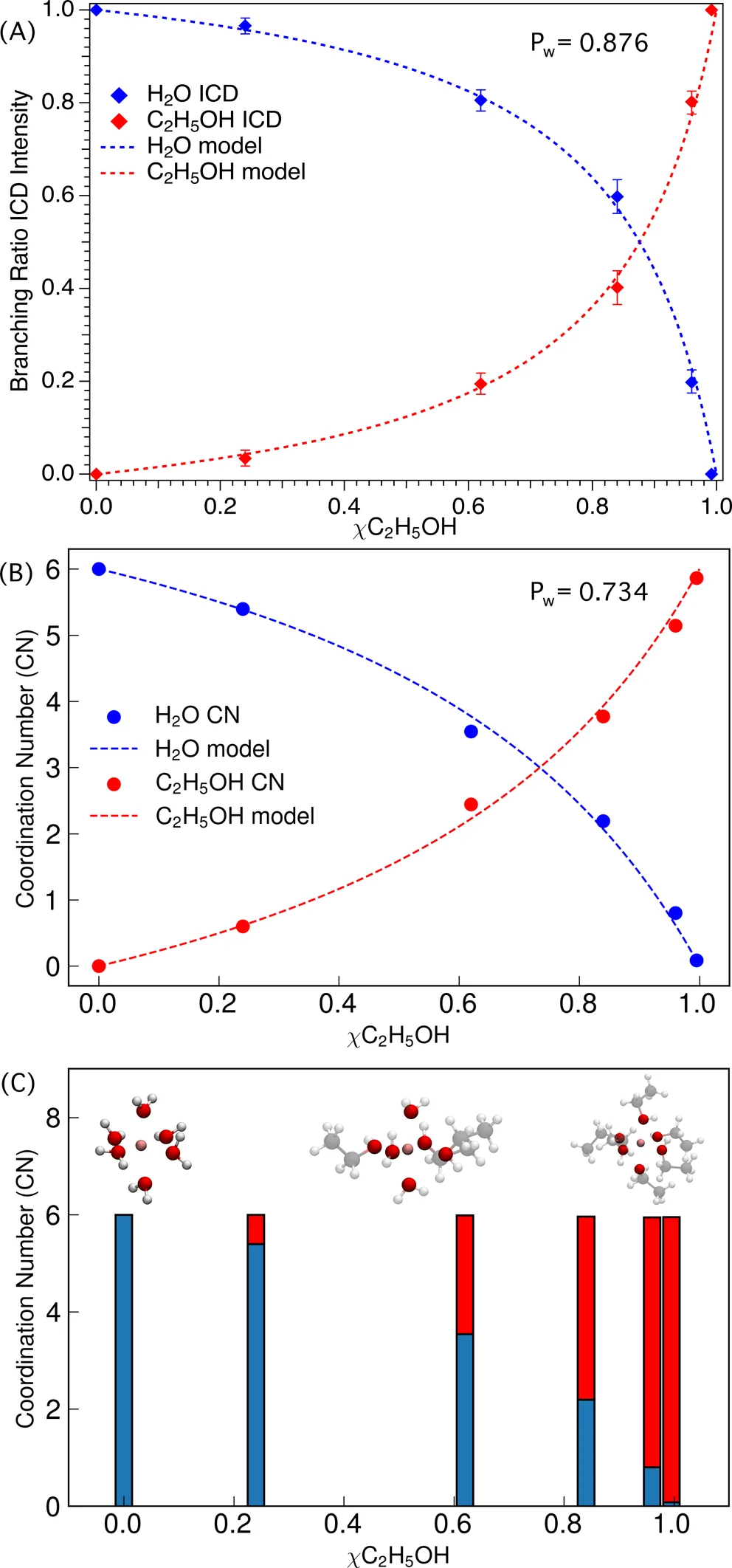
(A) Branching ratios of the water ICD peak area (blue symbols) and the scaled ethanol peak area (red symbols; see main text). The curves represent fits to a probabilistic adsorption model described in the main text (dashed blue: water; dashed red: ethanol), with *P*_w_ = 0.876. (B) Coordination numbers of Mg^2+^ ions obtained from MD simulations, plotted together with a fit using the same model (*P*_w_ = 0.734). (C) Histograms of the coordination numbers for each system, with representative first-solvation-shell structures extracted from the MD simulations shown above the bars.

In an aqueous solution, Mg^2+^ is expected to exhibit 6-fold coordination,^18,19^ but to the best of our knowledge, its solvation in ethanol has not been previously studied experimentally. Below, we show using MD simulations that this is also the case for ethanol and ethanol–water mixtures. However, even without making any assumptions about the solvation shell in the mixed case, a probabilistic description of the coordination can be employed. In the simplest possible model, we assume that the probability of a water or ethanol molecule occupying one of the coordination sites around the Mg^2+^ ion is given by a constant factor [*P*_w_ and (1 – *P*_w_), respectively] multiplied by the concentration of each constituent [(1 – χ_et_) and χ_et_, respectively], and normalized such that the total probability is unity

1 $$\begin{array}{l} P_{a}\left(\chi \right)=P_{w}\left(1-\chi_{\mathrm{et}}\right)/\left(P_{w}-2\chi_{\mathrm{et}}P_{w}+\chi_{\mathrm{et}}\right)\\ P_{b}\left(\chi \right)=\left(1-P_{w}\right)\chi_{\mathrm{et}}/\left(P_{w}-2\chi_{\mathrm{et}}P_{w}+\chi_{\mathrm{et}}\right) \end{array}$$

where *P*_a_ is the adsorption probability for water and *P*_b_ is the adsorption probability for ethanol. In this model, *P*_w_ is the probability that any coordination site around the Mg^2+^ ion is occupied by a water molecule in the case of equal concentrations of ethanol and water (χ_et_ = 0.5). If the probabilities for ethanol and water adsorption at each site were equal (*P*_w_ = 0.5), the occupation probabilities would be equal to the concentrations [*P*_a_ = (1 – χ_et_) and *P*_b_ = χ_et_]. In Figure 3A, the probabilities corresponding to the value *P*_w_ = 0.876, which gives the best least-squares fit to the data, are plotted. With this simple model, good agreement with the experimental data is obtained, suggesting that a water molecule is approximately 0.876/(1–0.876) ∼ 7 times more likely than an ethanol molecule to occupy any given coordination site in the solvation shell of the Mg^2+^ ion at equal mole fractions.

Another approach to understanding the composition of the solvation shell in the mixed cases is offered by MD simulations. Some representative structures obtained by MD are shown in Figure 3C, while Figure 4 shows the radial distribution functions (RDFs) around the Mg^2+^ ion of Mg^2+^–[O]­Water, Mg^2+^–[O]­Ethanol, and Mg^2+^–Cl^–^ for the mole fraction χ_et_ = 0.62. Plots of the RDFs for all mole fractions can be found in the Supporting Information (Figure S3).

**Figure 4:**
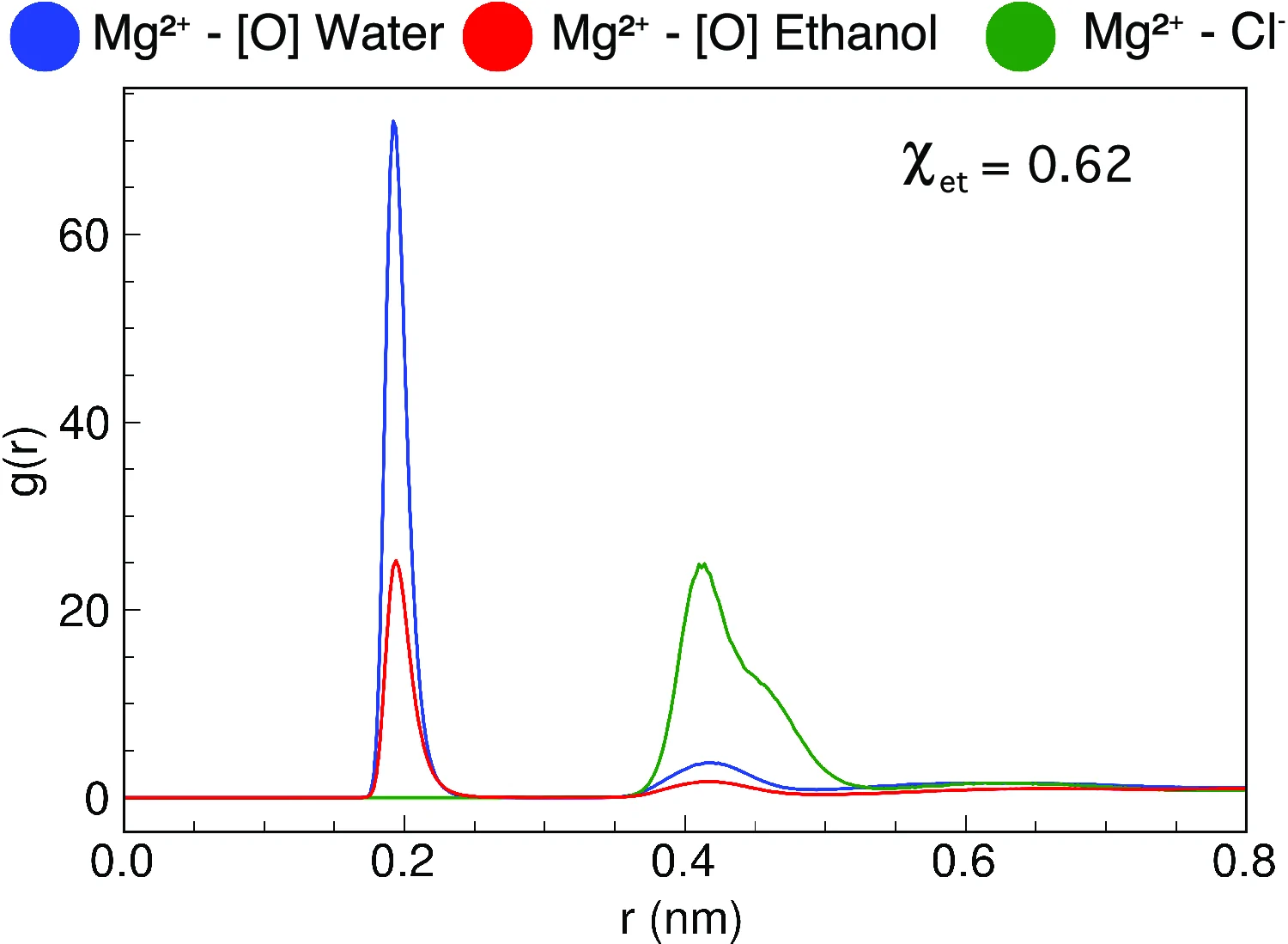
Radial distribution functions (RDFs) for the Mg^2+^–[O]­Water (blue curves), Mg^2+^–[O]­Ethanol (red curves), and Mg^2+^–Cl^–^ (green curves) interactions at an ethanol mole fraction of χ_et_ = 0.62.

As can be seen in Figure 4, both [O]­Water and [O]­Ethanol exhibit a sharp maximum at 0.2 nm, while Cl^–^ displays a broader maximum centered around 0.4 nm. Our MD simulations thus show that the first solvation shell around Mg^2+^ consists of water and ethanol molecules located at nearly the same distance from the Mg^2+^ ion. The coordination numbers shown in Figure 3B,3C were obtained by integrating each radial distribution function up to and including the first coordination shell, using the built-in rdf tool in the Gromacs package. From the integration of the RDFs, we find that the coordination number in the first solvation shell remains nearly constant at a value of 6, irrespective of the molar fraction of ethanol (see Figure 3C). These observations support the simple statistical model used for the analysis of the experimental data presented above. Water molecules exhibit a higher probability of being found in the first solvation shell than ethanol molecules. This is clearly evident for χ_et_ = 0.62 (Figure 4), where, despite ethanol being the dominant solvent, water remains more prevalent in the solvation shell.

The broad maximum for Cl^–^ around 0.4 nm indicates the formation of solvent-separated ion pairs, Mg^2+^–solvent–Cl^–^, where the solvent can be either a water or an ethanol molecule.

Focusing on the first solvation shell, Figure 3C shows the distribution of coordination environments found in the MD simulations at different bulk concentrations, illustrating the gradual substitution of water by ethanol as χ_et_ increases. Water is seen to persist in the solvation shell even at high ethanol concentrations (e.g., χ_et_ = 0.96), becoming virtually absent only at χ_et_ = 0.995. In Figure 3B, a least-squares fit of the coordination numbers derived from the MD simulations to the probabilistic model described above is shown. The results of the MD simulations are well described by this model and, when compared with the relative intensities obtained from the electron spectra, exhibit the same trend: a strong preference of Mg^2+^ ions for coordination with water. The value *P*_w_ = 0.734 indicates that water is approximately 0.734/(1–0.734) ∼ 3 times more likely than ethanol to coordinate with a Mg^2+^ ion at equal mole fractions.

To further investigate the structural changes in the solvation shell, the average Mg–O distances were analyzed as a function of ethanol mole fraction (Figure 5). While the Mg–O­(ethanol) distance follows a linear trend, the Mg–O­(water) distance exhibits a nonlinear behavior. This suggests that the water solvation environment is sensitive to the bulk solvent composition, likely due to changes in the hydrogen-bond network and packing density. Interestingly, the intersection of the water and ethanol distance trends correlates with the crossover point observed in the coordination number plots, see Figure 3B.

**Figure 5:**
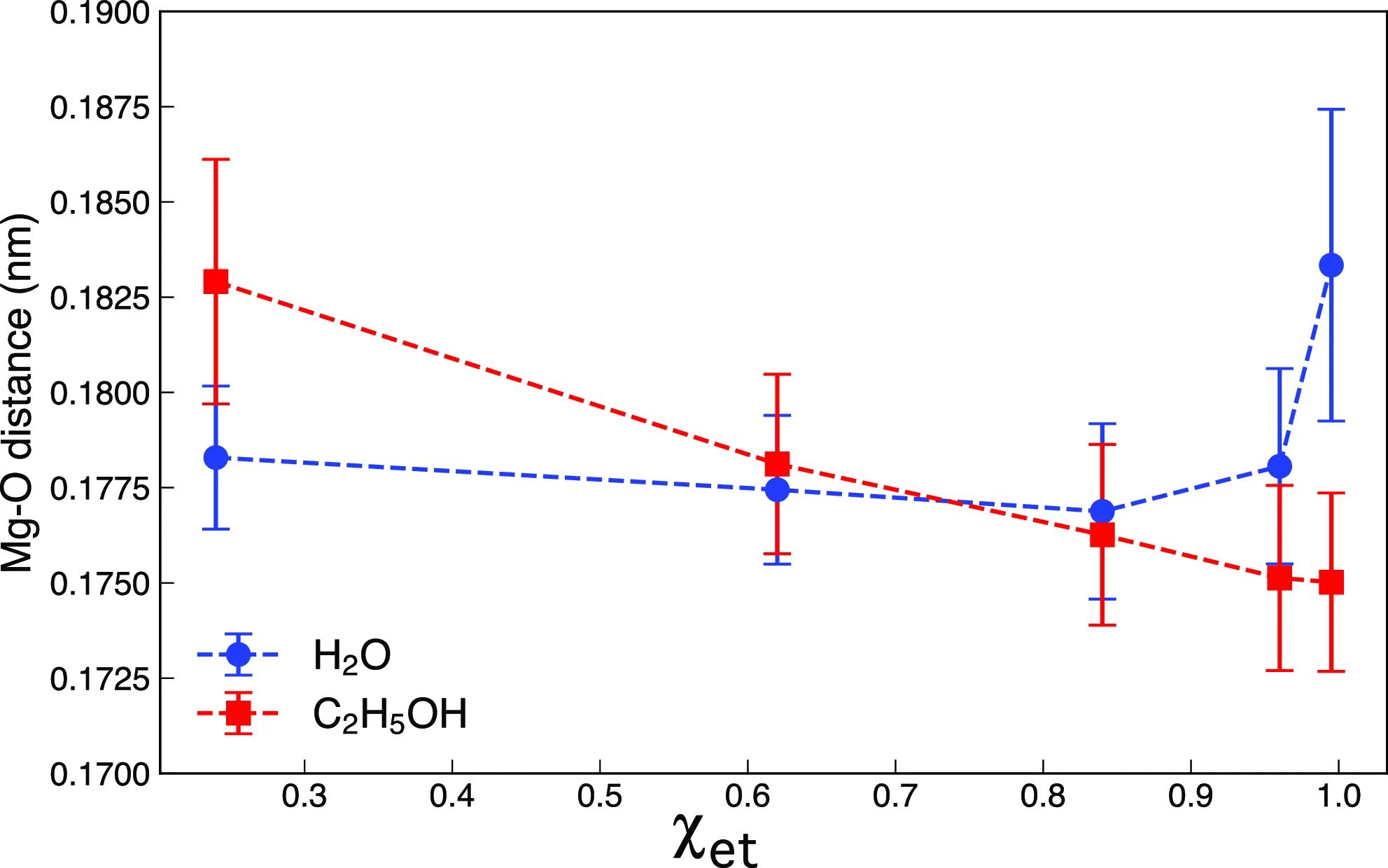
Average Mg–O distances as a function of ethanol mole fraction derived from MD simulations. The ethanol distance (red squares) follows a linear trend, whereas the water distance (blue circles) exhibits nonlinear behavior. The dashed lines are included as guides to the eye. Error bars represent the standard deviations of the distance distributions.

The observed strong preference for water in the first solvation shell raises the question of the driving force behind this selectivity, particularly given the specific interaction potentials. Our analysis of the Lennard-Jones (LJ) parameters reveals that the short-range attraction is marginally stronger for ethanol (σ_*O*–*Mg*_ = 0.17988 nm) than for water (σ_O–Mg_ = 0.18100 nm). However, this short-range advantage is overcome by electronic and steric factors. Electronically, water possesses a substantially larger dipole moment (∼1.85 D) than ethanol (∼1.69 D), favoring its ion-dipole interaction with the Mg^2+^ cation.

Structurally, the hydrogen-bond network of water plays a critical role. Water molecules form a highly organized network near the ion; creating a cavity in this network large enough to accommodate the bulky ethyl group of an ethanol molecule is energetically unfavorable. Thus, steric hindrance and the penalty of disrupting the local H-bond network limit ethanol’s involvement. This effect is starkly illustrated in the simulation with the highest ethanol concentration (χ_et_ = 0.995): despite the scarcity of water (only ten molecules present), 52% of these water molecules were found interacting with Mg^2+^ ions, confirming a robust thermodynamic driving force for aqueous solvation. Similar complex behavior in ethanol–water mixtures at high concentrations has previously been discussed in the context of azeotrope formation.^26^

## Conclusion

Using intermolecular Coulombic decay (ICD) of Mg 1s vacancies and molecular dynamics simulations, we investigated the microsolvation of Mg^2+^ ions in ethanol–water mixtures. The experimental ICD spectra show a clear evolution in shape and a 20% decrease in total intensity as the solvent changes from pure water to pure ethanol. No ICD signal from Cl^–^ ions was detected in any of the solutions, a clear indication that contact ion pairs are rare at the chosen concentration of MgCl_2_ (0.5 M). A quantitative analysis of the spectral components reveals a strong preferential solvation of Mg^2+^ by water molecules, even in ethanol-rich solutions. This observation is well-described by a simple probabilistic model, which indicates that water is around seven times more likely than ethanol to occupy a coordination site. These experimental findings are strongly supported by our MD simulations, which show that the steric hindrance and energy penalty of disrupting the local H-bond network from ethanol coordination make water dominate the first solvation shell across a wide range of concentrations.

This work demonstrates the power of core-level ICD as a site-specific probe, combining chemical selectivity and local sensitivity to elucidate the local environment of ions in complex liquid mixtures, providing information about the identities of the species in the first solvation shell and their respective coordination numbers, pointing toward applications in, e.g., batteries and systems of environmental and biological relevance.

## Methods

### Experimental Section

#### Sample Preparation

Solutions of 0.5 M (mol·dm^–3^) MgCl_2_ were prepared by adding a given mass of salt in a defined mass of ethanol and then adding water to reach the volume corresponding to the desired concentration and weighing the amount of water added. For the solution with the highest concentration of ethanol, the mass of the solvent was unfortunately not measured, and the ethanol concentration for this solution is an estimate from known densities. For solutions with lower ethanol concentration, MgCl_2_ hexahydrate was used (MgCl_2_·6H_2_O, Sigma-Aldrich, ≥99.0% purity), and for the two highest concentrations of ethanol, anhydrous MgCl_2_ was used (MgCl_2_, Carl Roth, ≥99.0% purity dry substance, ≤2.0% water). All mixtures were prepared with 99.8% ethanol (Carl Roth, Rotipuran, ≥99.8% purity, ≤0.2% water) and deionized water (Milli-Q, 18.2 MΩ·cm). The masses of all compounds and the concentrations for all solutions are presented in Table S2 in the Supporting Information.

#### Electron Spectroscopy

The data presented were obtained at the P04 beamline of the synchrotron facility PETRA III, DESY, Hamburg, Germany,^27^ with a photon energy of ∼1319 eV. The beamline exit slit was 150 μm, together with a 1200 l/mm grating, giving a photon bandwidth of ∼0.4 eV, and the focal spot size at the interaction region was approximately 180 μm (horizontal) × 50 μm (vertical). The photon energy was chosen to be above the Mg 1s ionization threshold, but close enough to have a high cross section for 1s photoionization, to obtain sufficient signal from the relatively weak ICD channel. Photon and kinetic-energy calibrations were performed for the aqueous case, using published values for the kinetic energy of the Mg KL_2,3_L_2,3_ features^11,21^ and the binding energy of the Mg 2s photoelectron line.^10^ The photon energy for the other cases may vary slightly, which is why we have chosen, for illustrative purposes, to present the spectra on a common kinetic-energy scale, where we have set the kinetic energy of the Mg KL_2,3_L_2,3_^1^S peak to the same for all spectra.

Data were obtained using a liquid microjet sample introduction system and the EASI (Electronic structure of Aqueous Solutions and Interfaces) liquid-jet photoemission setup.^28^ The jets were formed by a quartz capillary with an inner diameter of 36 μm, using a flow rate of 1.0 mL/min. During operation, the pressure in the main experimental chamber was maintained at ∼5 × 10^–4^ mbar. The spectrometer, equipped with an 800 μm skimmer, was mounted with the lens at 130° with respect to the photon-beam propagation direction, i.e., in a near-magic-angle configuration, minimizing any angular distribution effects on the intensity. The spectrometer was operated with a pass energy of 200 eV, and the entrance slit of the spectrometer had a width of 0.8 mm, giving a spectrometer resolution of ∼0.4 eV.

The spectra were fitted to obtain the integrated areas of all relevant features. Details regarding the fitting procedure, example fits, and the results can be found in the Supporting Information.

### Molecular Dynamics

The interactions of water and MgCl_2_ were described using the TIP4P-2005 model together with parameters derived by Vega and co-workers,^29^ and OPLS (Optimized Potentials for Liquid Simulations)^30^ was used to describe ethanol. We empirically modified the Lennard-Jones parameters for ethanol to match the experimental results, since ion pairing was still observed in systems with higher ethanol fractions. Packmol^31^ was used to build the simulation box for each system, which consisted of 2000 water and ethanol molecules, following the experimental ethanol mole fractions (χ_et_). The ideal volume was used to add MgCl_2_ until a concentration of 0.5 mol·L^–1^ was reached.

Gromacs 2023.3 ^32^ was used for all molecular dynamics (MD) simulations. First, an energy minimization was performed using the steepest descent algorithm, coupled with the conjugate gradient method every 10 steps. Then, an equilibration step of 2 ns was performed, followed by an 8 ns production run, both in the NPT ensemble at 298.15 K and 1 bar. Temperature control was maintained using a v-rescale thermostat^33^ with a coupling time of 0.1 ps, while pressure was controlled semi-isotropically using the c-rescale barostat^34^ with a coupling time of 1 ps.

Particle mesh Ewald (PME)^35^ was used for Coulomb interactions, with a 1.2 nm cutoff applied to all interactions. The fourth order LINCS constraint algorithm^36^ was used for bonds involving hydrogens. Minimum distances between oxygen atoms and Mg^2+^ ions were calculated using the gmx pairdist tool.

The Lennard-Jones parameters used for the O–Mg interaction were σ_O–Mg_ = 0.18100 nm for water and σ_O–Mg_ = 0.17988 nm for ethanol. The ϵ values were slightly smaller for ethanol, indicating that ethanol oxygen atoms exhibit an approximately 1% stronger short-range interaction with Mg compared to water.

Gromacs tools, Trajectory Analyzer and Visualizer (TRAVIS),^37^ Visual Molecular Dynamics (VMD),^38^ and internal scripts were used for visualization and analysis of MD results. More details about the validation of the models and postprocessing analysis are described in the Supporting Information.

## Supplementary Material



## Notes

Details of the fitting procedure for the experimental data; tabulated data for peak areas obtained from the spectra; a discussion of the occurrence of contact ion pairs in the solutions; tabulated data for the compositions of the solutions used in the experiments; a discussion of the influence of inelastic scattering on the intensities of Auger and ICD features; details regarding the molecular dynamics simulations; plots of potentials, radial distribution functions, mean-square displacements, diffusion coefficients, and the probability density distribution of the minimum O–Mg^2+^ distances obtained from the MD simulations (PDF)
