# Supplementary material for: Preferential Solvation of Mg2+ in Ethanol–Water Mixtures Probed by Intermolecular Coulombic Decay and Molecular Dynamics Simulations
Source: ACS Phys Chem Au. 2026 Aug 6;6(5):1062–9. doi: 10.1021/acsphyschemau.6c00039 (PMC13614083; doi:10.1021/acsphyschemau.6c00039)
Supplement: Supplementary file 1 [file pg6c00039_si_001.pdf]

Supporting Information: Preferential Solvation of Mg^{2+} in Ethanol-Water Mixtures Probed by Intermolecular Coulombic Decay and Molecular Dynamics Simulations

Ouassim Hocine Hafiani,[†] Alexandre M. Pereira,[‡] Luciano T. Costa,[‡] Michele Pugini,[¶] Shirin Gholami,[¶] Nicolas Velasquez,[¶] Florian Trinter,[¶] Iyas Ismail,[§] Ricardo Marinho,[∥,⊥] Alexandra Mocellin,[∥] Arnaldo Naves de Brito,[#] Carl Caleman,[†] Lukáš Tomaník,[@] Bernd Winter,[¶] Olle Björneholm,[†] and Gunnar Öhrwall[*,△]

†*Department of Physics and Astronomy, Uppsala University, Uppsala, 75120, Sweden*
‡*MolMod-CS, Institute of Chemistry, Fluminense Federal University, Niterói, 24020-141, Brazil*
¶*Fritz-Haber-Institut der Max-Planck-Gesellschaft, Berlin, 14195, Germany*
§*Sorbonne Université, CNRS, Laboratoire de Chimie Physique-Matière et Rayonnement, Paris, 75005, France*
∥*Institute of Physics, Brasilia University, Brasília, 70919-970, Brazil*
⊥*Institute of Physics, Federal University of Bahia, Salvador, 40170-115, Brazil*
#*Department of Applied Physics, Institute of Physics Gleb Wataghin, Campinas University, Campinas, 13083-859, Brazil*
@*Department of Analytical Chemistry, University of Chemistry and Technology, Prague, 16628, Czech Republic*
△*MAX IV Laboratory, Lund University, Lund, 22100, Sweden*

E-mail: gunnar.ohrwall@maxiv.lu.se
Phone: +46-(0)46-2224665

Experimental details and considerations

Fitting details

The electron spectra were fitted using Igor Pro[S1] and the SPANCF curve fitting macro package, presented in Refs.[S2,S3] Symmetric Voigt profiles were used for all peaks. The Lorentzian lifetime widths were fixed to the recommended values from Ref.,[S4] 0.46 eV for the Mg 2s peaks, and to 0.334 eV for the features originating from Mg 1s ionization (KLL Auger features and ICD features). In the fits of the ICD features, a linear background and a broad Gaussian peak centered around the kinetic energy of ∼1248 eV (to account for the inelastic loss structures from O 2s photoionization in the spectrum) were included. The width and separation from the Mg 2s peak of this Gaussian feature were kept constant for all spectra. The statistical uncertainties for the peak areas were derived from the standard deviation of the fitting parameters, which is estimated from the curvature matrix of the χ^2 surface in the SPANCF fitting package, and are presented in Figure 2B as 1.96 * (compound error). The error bars for the branching ratios

(Figure 3A) were calculated from the extreme values of the areas when taking these errors into account.

Example fits for three different cases: pure water ($\chi_{et} = 0$), an ethanol-water mixture ($\chi_{et} = 0.84$), and (nearly) pure ethanol ($\chi_{et} = 0.99$) are presented in Figure 2A of the main manuscript. The shape of the ICD features for water was modeled with three peaks for states where the outer-valence orbitals of water are involved (around 1235-1240 eV) and one peak for the O 2s (around 1222 eV). This picture is consistent with the model used in Ref.,[S5] where the peaks are associated with the outer-valence states $1b_1$, $3a_1$, and $1b_2$ of water. Similarly, we modeled the ICD in ethanol with one peak for O 2s contributions (around 1222 eV), two peaks for C 2s contributions (around 1229 eV and 1233 eV), and four peaks for outer-valence contributions (around 1235-1240 eV). The outer-valence spectrum of liquid ethanol has seven states,[S6] which overlap substantially and are not easily assigned, and therefore we used only four peaks, as a minimum to acceptably fit the outer-valence ICD features of ethanol. We do not attribute any meaning to the peaks; we used them as a practical means to fit the spectra and only considered the total intensity of the ICD features. The Gaussian widths of all these features were free parameters, but they were constrained to have the same value for all spectra. In the same way, the intensities were free parameters, but the relative intensities of all peaks were constrained to be equal in all spectra, so only the total intensity differed between spectra. In the mixed cases, the ICD was thus modeled with the sum of the water and ethanol ICD shapes, where only the intensity was allowed to vary, as illustrated for the case $\chi_{et} = 0.84$ in Figure 2A. The total areas of the peaks determined by the fitting procedure are presented in Table S1.

Table S1: Peak areas for the ICD features from water and ethanol in arbitrary units, for varying molar fraction of ethanol (χ_{et}), obtained from the fitting procedure described above.

χ_{et}	water	ethanol	total
0	1	0	1
0.24	0.948	0.026	0.975
0.62	0.747	0.144	0.891
0.84	0.554	0.297	0.852
0.96	0.203	0.655	0.858
0.99	0	0.797	0.797

Ion pairing

In aqueous solutions of $MgCl_2$ at moderate concentrations, contact ion pairs are not expected to form, as demonstrated by dielectric relaxation spectroscopy[S7] and molecular dynamics (MD) simulations.[S8,S9] However, constrained MD simulations of $MgCl_2$ solvation in mixed water-ethanol solutions[S10] revealed clear minima in the potentials of mean force for the Mg^{2+}–Cl^- ion pair within the first solvation shell for all solvent compositions, including neat water. The depth of the minima, located at an ion separation of approximately 2.3 Å, increased with mole fraction of ethanol, suggesting a higher propensity for contact ion pair formation in ethanol-rich environments. To the best of our knowledge, no experimental evidence concerning the presence of contact ion pairs in these specific mixtures exists.

The kinetic energy of the Cl 3p contribution to the ICD signal can be approximately estimated for the aqueous case by subtracting the known binding energies of the two holes in the final state from the energy of the Mg 1s hole: E_b(Mg 1s) − E_b(Mg 2p) − E_b(Cl 3p) = (1309.9 − 55.6 − 9.6) eV = 1244.7 eV.[S11–S13] For ICD involving water orbitals, it has often been found that such a simple estimate is close to experimentally observed values, with a small difference on the order of a few eV that can be attributed to Coulomb repulsion between the metal ion and the ionized water molecule. The neutral final state of the Cl^- ion after ionization would result in lower Coulomb repulsion than for water, where both ions are positively charged after ionization, and the corresponding spectral intensity would therefore be expected to appear close to this energy. Calculations of the microsolvation of potassium ions from KCl

by water molecules[S14] show that the chloride counterion would contribute significantly to the ICD spectrum if the metal cation were not fully solvated by water. Although the cation and, in most cases, the solvent differ from those considered here, we interpret the low spectral intensity of the ICD feature in the region where the Cl 3p contribution is expected as evidence that contact ion pairs are rare in both water and ethanol.

Data for solutions used in measurements

The masses and concentrations of the solutions used in the measurements are presented in Table S2. The ethanol concentrations were calculated using the assumption of a water content of 0.2% in the ethanol and a water content of 2% in the anhydrous $MgCl_2$, the purities of the compounds stated by the supplier. The water content in $MgCl_2$ hexahydrate was assumed to be nominal, six water molecules per $MgCl_2$.

The influence on spectral intensities from IMFP differences

A difference in the kinetic-energy dependence of the inelastic mean free path (IMFP) could in principle lead to a change in the relative intensity of the Mg KLL features (with a kinetic energy of ∼1170 eV) and the $KL_{2,3}$ ICD features (∼1240 eV), when changing the solvent. The IMFP of liquid water in this kinetic-energy region is estimated to be of the order ≈4.3-4.5 nm[S15]. The IMFP for electrons in ethanol has not been measured or calculated to the best of our knowledge. However, as in water, a kinetic energy of ∼1.2 keV is well above the deepest core-ionization energy (O 1s at ≈0.54 keV) for the constituent atoms of the solvent, and since the electron density of ethanol is lower than that of water (0.33 $Å^{-3}$ and 0.27 $Å^{-3}$ for water and ethanol, respectively), we expect the IMFP to be slightly longer for ethanol in this kinetic-energy range.

Empirical models for the kinetic-energy dependence of the electron IMFP in condensed matter often rely on power laws. For example, Seah and Dench[S16] express the IMFP(λ) as $\lambda = \mathrm{a} \cdot E_k^{-2} + \mathrm{b} \cdot \sqrt{E_k}$, where E_k is the kinetic energy of the electron and a and b are constants. The first, inversely quadratic term in the expression above is negligible at the energies considered here, which means that in this model, the IMFPs in ethanol and water are expected to show a $\sqrt{E_k}$ dependence and only differ by a multiplicative factor. At the energies in question (1170-1240 eV), the IMFP would then be expected to increase by ≈3% from the lowest kinetic energy to the highest *for both cases*. Therefore, we conclude that no significant difference in relative intensity can be attributed to differences in IMFP alone.

Since both the KLL and ICD spectral features originate from the same species in the solution (Mg^{2+} ions), the distribution of the ions, which may well be different for water and ethanol solutions, will affect the intensities of the Mg $KL_{2,3}L_{2,3}$ ^1S and $KL_{2,3}$ ICD features in the same way. MD simulations of the liquid-vapor interface of aqueous $MgCl_2$ solutions show that the Mg^{2+} ions in aqueous $MgCl_2$ avoid the surface, but that a substantial amount of ions are present already 5 Å below the surface, and that bulk density is reached around ≈2 nm below the surface.[S8] With the IMFP ≈ 4.3-4.5 nm, a substantial part of the observed intensity thus comes from the bulk of the liquid and the experiment is relatively insensitive to variations in the outermost surface region.

Using the depth distribution of the Mg^{2+} concentration in water[1], and assuming an exponential intensity decrease as a function of the distance from the surface, we can estimate how the relative intensity of the KLL and ICD features, with kinetic energies of 1170 eV and 1240 eV, respectively, would differ between water and ethanol, if we make an assumption about the IMFP and the depth distribution in ethanol. For a case with an IMFP in ethanol equal to that in water and an unrealistic density of ethanol that is zero down to 4 nm below the surface and has the bulk value below 4 nm, we estimate that scaling the ICD feature (at ∼1240 eV) to the KLL feature (at ∼1170 eV)

[1]Digitized from Figure 3a in Ref.[S8]

Table S2: Masses of contents and obtained concentrations in the used solutions. Masses in g, volume in l, concentration $MgCl_2$ in M ($mol \cdot dm^{-3}$), concentration ethanol in mol%.

M $MgCl_2$	M $MgCl_2 \cdot 6H_2O$	M H_2O	M C_2H_5OH	Vol.	C $MgCl_2$	χ_{et}
	50.83	476.1	0	0.5	0.50	0
	50.83	220.3	203.1	0.5	0.50	24.2
	50.86	51.0	327.8	0.5	0.50	61.9
	50.83	0	373.0	0.5	0.50	84.0
23.82		5.3	390.1	0.5	0.49	95.9
18.84		0	-	0.4	0.48	99.2

would introduce a ≈4% difference between the water and ethanol cases. For a more realistic depth distribution, the difference will be even smaller, and we conclude that differences in the IMFP and depth distribution will at most have marginal effects on our observations.

Atomistic simulations for water-ethanol mixtures

Molecular models

As a rule, simple salt crystals dissolve in water at low to moderate salt concentrations. However, several studies have reported anomalous behavior for such systems in molecular dynamics simulations, where salt crystals and ion pairs form below the saturation limit when using different types of water and ion models.[S17] A common approach to hindering the formation of ion pairs is to scale the ion charge,[S5] but this is not always sufficient. Vega et al.[S18] not only scaled the charges but also adjusted the Lennard-Jones (LJ) parameters for ion-ion and ion-water interactions, leading to a satisfactory description of a range of ion-solvation properties. Recently, Boyn and Carter used multilevel quantum mechanics/molecular dynamics simulations to investigate the pH-dependent dehydration dynamics of Mg and Ca cations in aqueous solutions.[S9]

As mentioned in the main text, we used the Vega parameters[S18] and the TIP4P-2005 model for the water interactions with $MgCl_2$, and OPLS[S19] to describe ethanol. Using this approach, the formation of ion pairs was still observed in systems with higher ethanol fractions, and we empirically modified the Lennard-Jones parameters for ethanol to match the experimental results. Figure S1 shows the LJ parameters before and after this empirical modification.

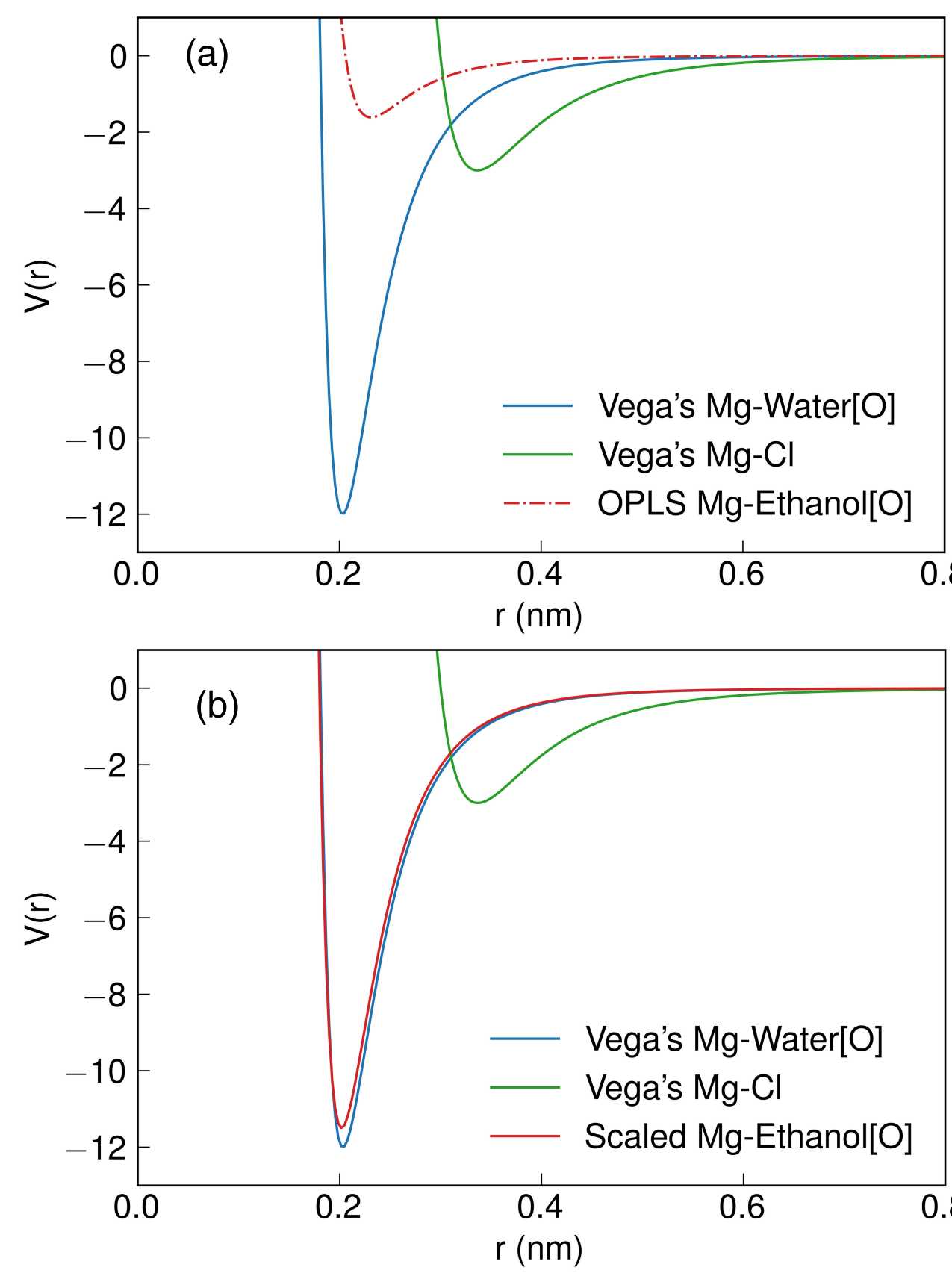

Figure S1: Lennard-Jones fitting to reproduce the experimental data for Mg-ethanol interactions following the same procedure employed by Vega[S18] to adjust the classical force field parameters for Mg-water interactions.

To avoid arbitrary scaling of the parameters, we adjusted the ethanol[O]-(Mg/Cl) interactions on the basis of the Vega pair potentials. First, we calculated the values for all parameters assuming the use of Lorentz-Berthelot combination rules, measuring how much the ethanol parameters differ from those of water. The σ values were approximately 99% and the ϵ values were 95% of the corresponding water values. We then used parameters reported previously by Vega to scale the OPLS ethanol model. The results are shown in Figure S1(b).

The new non-bonded parameters for Mg–ethanol interactions are $\sigma = 0.17988\,\mathrm{nm}$ and $\epsilon = 11.49679\,\mathrm{kJ\,mol^{-1}}$. For Cl, the values are $\sigma = 0.42125\,\mathrm{nm}$ and $\epsilon = 0.05938\,\mathrm{kJ\,mol^{-1}}$.

Computational methods

Packmol[S20] was used to build the simulation box for each system, which was composed of 2000 molecules (water + ethanol), following the experimental mole fractions of ethanol (χ_{et}). The simulation volume was chosen such that the $MgCl_2$ concentration was 0.5 mol·L^{-1}. Table S3 presents the mole fractions of ethanol and the corresponding number of Mg^{2+} ions added.

Table S3: Number of Mg^{2+} ions in each system.

χ_{et}	# Mg^{2+}
0	18
0.24	27
0.62	43
0.84	51
0.96	56
0.995	58

Water (TIP4P-2005) and its interactions with Mg^{2+} and Cl^- ions were described following Vega et al.[S18] The structure and parameters of OPLS[S19] ethanol are available on the Virtual Chemistry website.[S21,S22] Only the pair potentials between the oxygen of ethanol and Mg^{2+} and Cl^- were empirically modified, as previously described. Figure S2 shows the fit between Vega's Mg^{2+} - Water[O] interactions and the $\chi_{et} = 0$ system. This result is also in good agreement with a previous study of Mg^{2+} solvation in aqueous solutions (see Figure S4 in the Supplementary Information of this work).[S5]

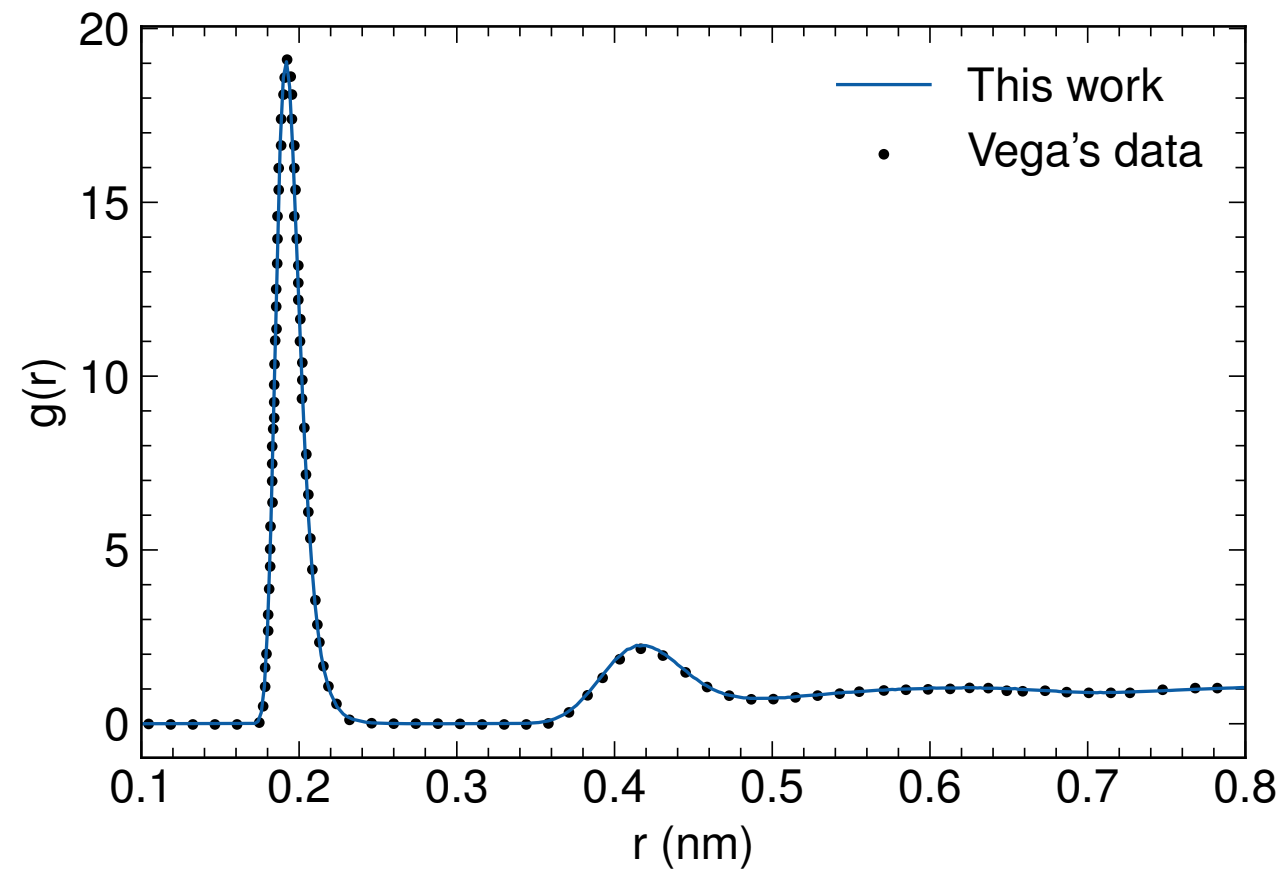

Figure S2: Comparison of our fitted potential results with Vega's Mg^{2+} - Water[O] radial distribution function for the neat water system, $\chi_{et} = 0$.

RDFs for all ethanol mole fractions

In Figure S3, the radial distribution functions and integrated number of particles obtained from the simulations are presented.

The mean-square displacement (MSD) was computed using the *gmx msd* tool in GROMACS. Diffusion coefficients were extracted from the linear regime of the MSD using the Einstein relation.[S23] Figure S4 presents the MSD curves for Mg^{2+} and Cl^- ions at different ethanol mole fractions, while the corresponding diffusion coefficients are shown in Figure S5.

Our results highlight the reliability of the proposed model when compared with the data reported by Chatterjee et al.,[S10] who observed higher diffusion coefficients for both ions. It is important to note that their study reported the formation of ion pairs, in contrast to the behavior observed in our simulations and experiments. Since Mg^{2+} ions remain strongly coordinated within their solvation shell, lower diffusion coefficients are expected in our systems.

Interestingly, we observe a slight decrease in ionic diffusion in the pure ethanol system,

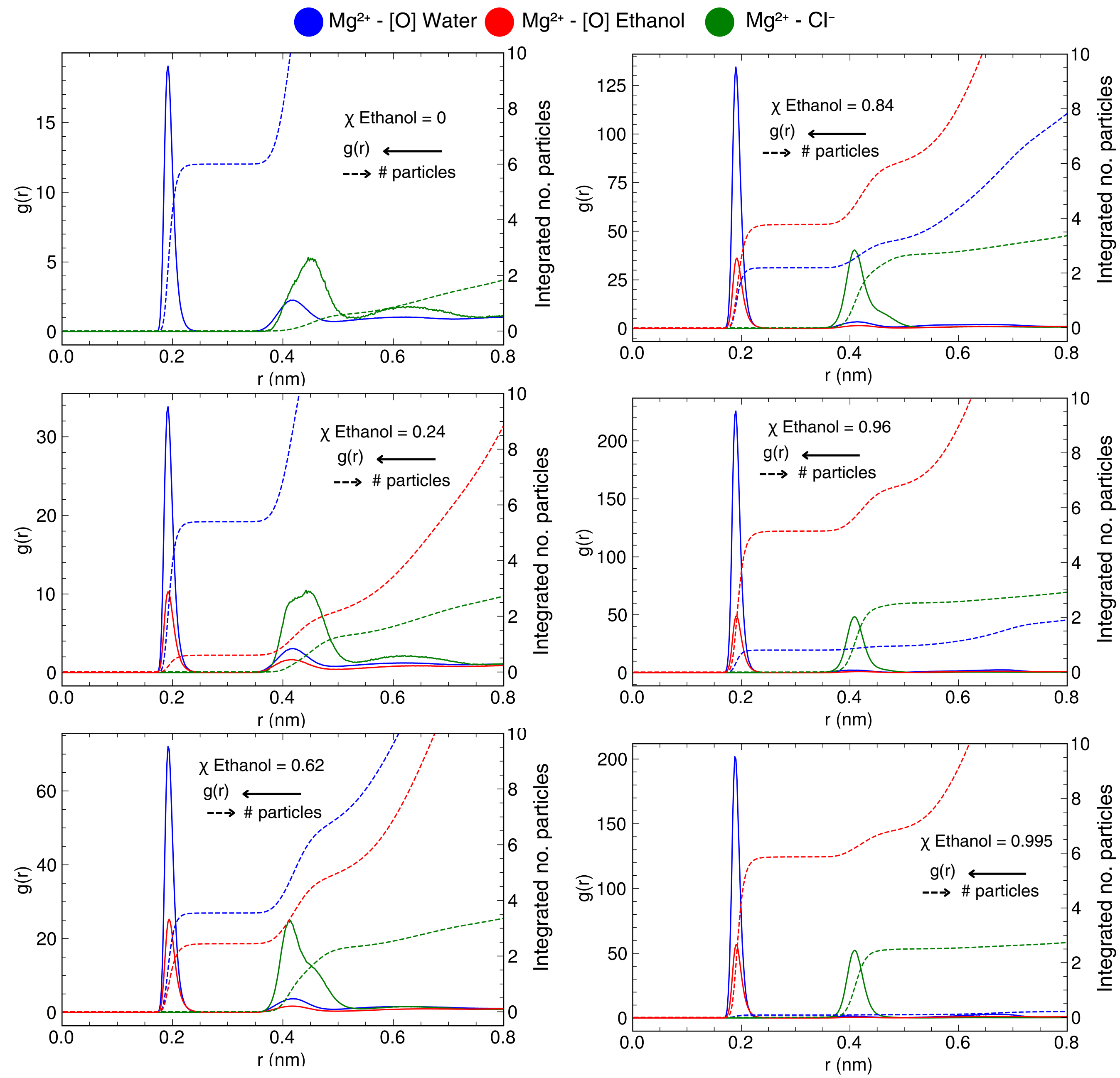

Figure S3: Radial distribution functions (RDFs) and integrated number of particles for Mg^{2+} - [O]Water (blue curves), Mg^{2+} - [O]Ethanol (red curves), and Mg^{2+} - Cl^{-} (green curves) interactions for all mole fractions of ethanol.

which contrasts with the trend reported by Chatterjee et al.,[S10] who found an increase in diffusion from a mole fraction of 0.9 to pure ethanol.

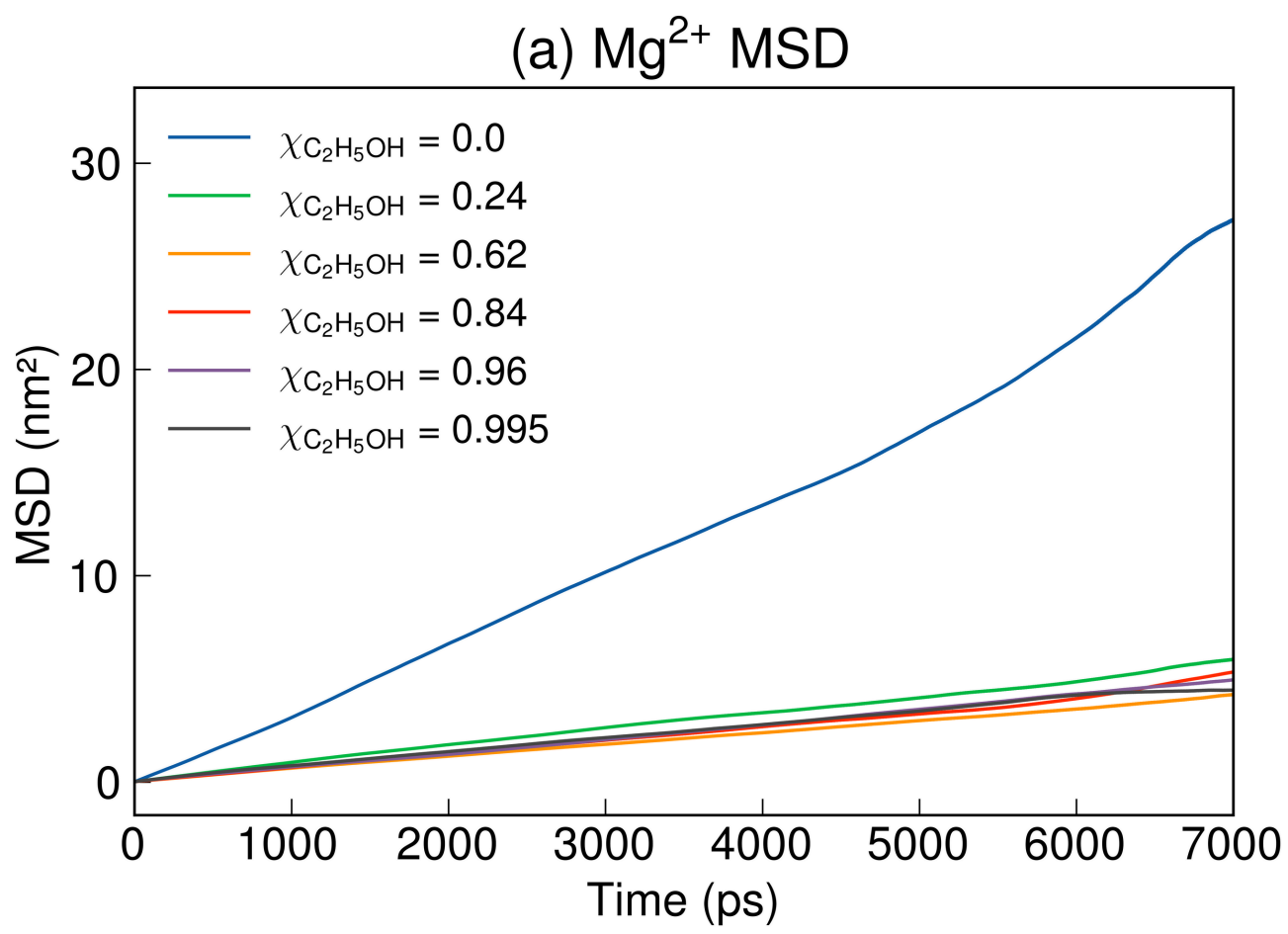

Figure S4: Mean-square displacements (MSDs) for the Mg^{2+} (a) and Cl^{-} (b) ions from the MD simulations over the mole fractions of ethanol.

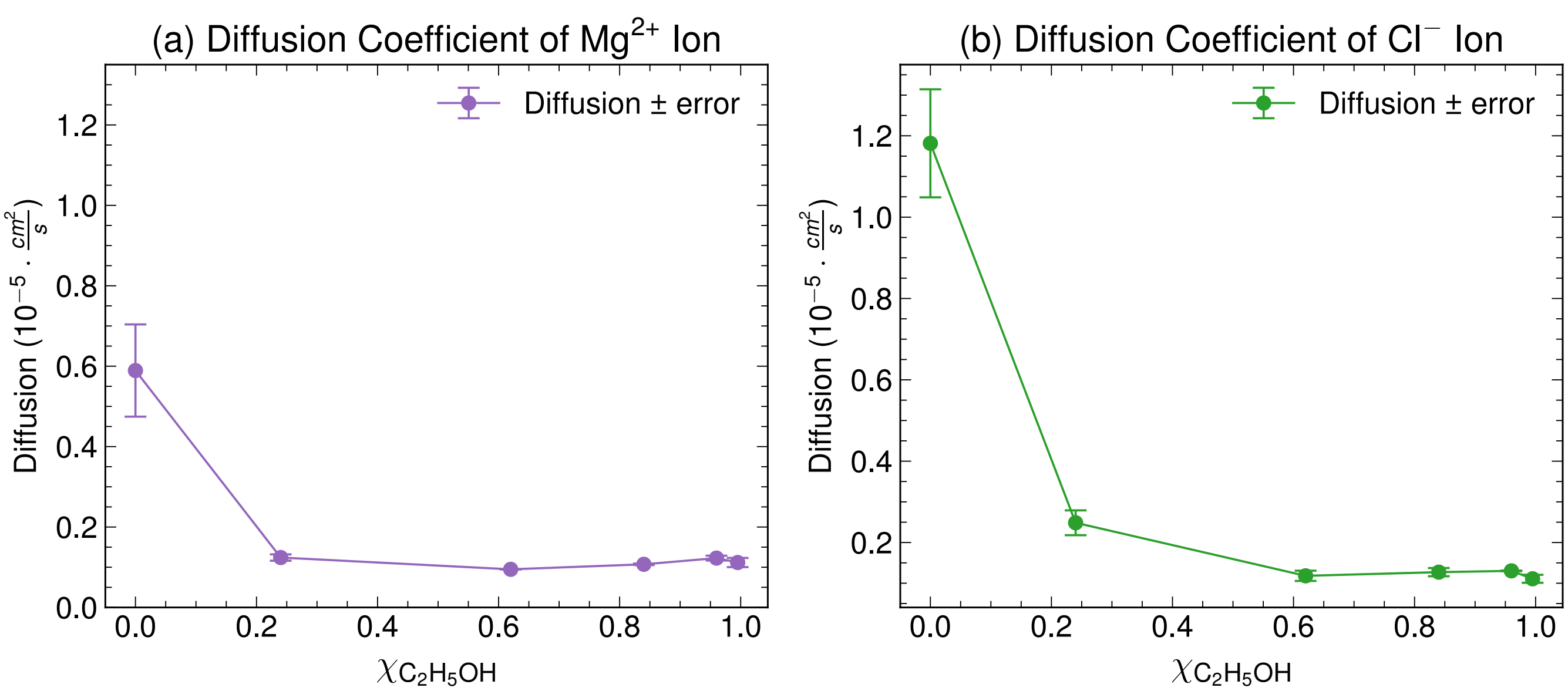

Figure S5: Calculated diffusion coefficients for the Mg^{2+} (a) and Cl^{-} (b) ions from the MD simulations versus mole fraction of ethanol.

Probability distribution of Mg–O distances

To further investigate the structural arrangement within the first solvation shell, we calculated the minimum distance between oxygen atoms (of both water and ethanol) and Mg^{2+} ions using the `gmx pairdist` tool. Figure S6 presents the probability distribution of these distances across all ethanol fractions. The data were fitted with normal distribution functions (dotted lines).

This analysis reveals the subtle interplay between short-range and long-range interactions. The Lennard-Jones (LJ) parameters used for the O-Mg interaction (σ_{O-Mg} = 0.18100 nm for water and σ_{O-Mg} = 0.17988 nm for ethanol) actually result in a slightly stronger (∼1%) short-range interaction for ethanol. However, water possesses a significantly larger dipole moment (∼1.85 D) compared to ethanol (∼1.69 D).

As shown in Figure S6, water preferentially occupies the first solvation shell up to $\chi_{et} \approx 0.84$. In the $\chi_{et} = 0.24$ system, ethanol molecules are statistically found farther from the metal center than water. This is attributed to the hydrogen-bond network: water forms a tight network around the Mg^{2+} ion, and creating a cavity large enough to accommodate the bulky ethyl group of an ethanol molecule is energetically unfavorable. Consequently, steric hindrance and network stability override the slightly favorable short-range LJ potential of ethanol. Even at $\chi_{et} = 0.995$, where only ten water molecules are present in the simulation, a distinct peak for water-Mg coordination is observed, confirming the strong preference for water.

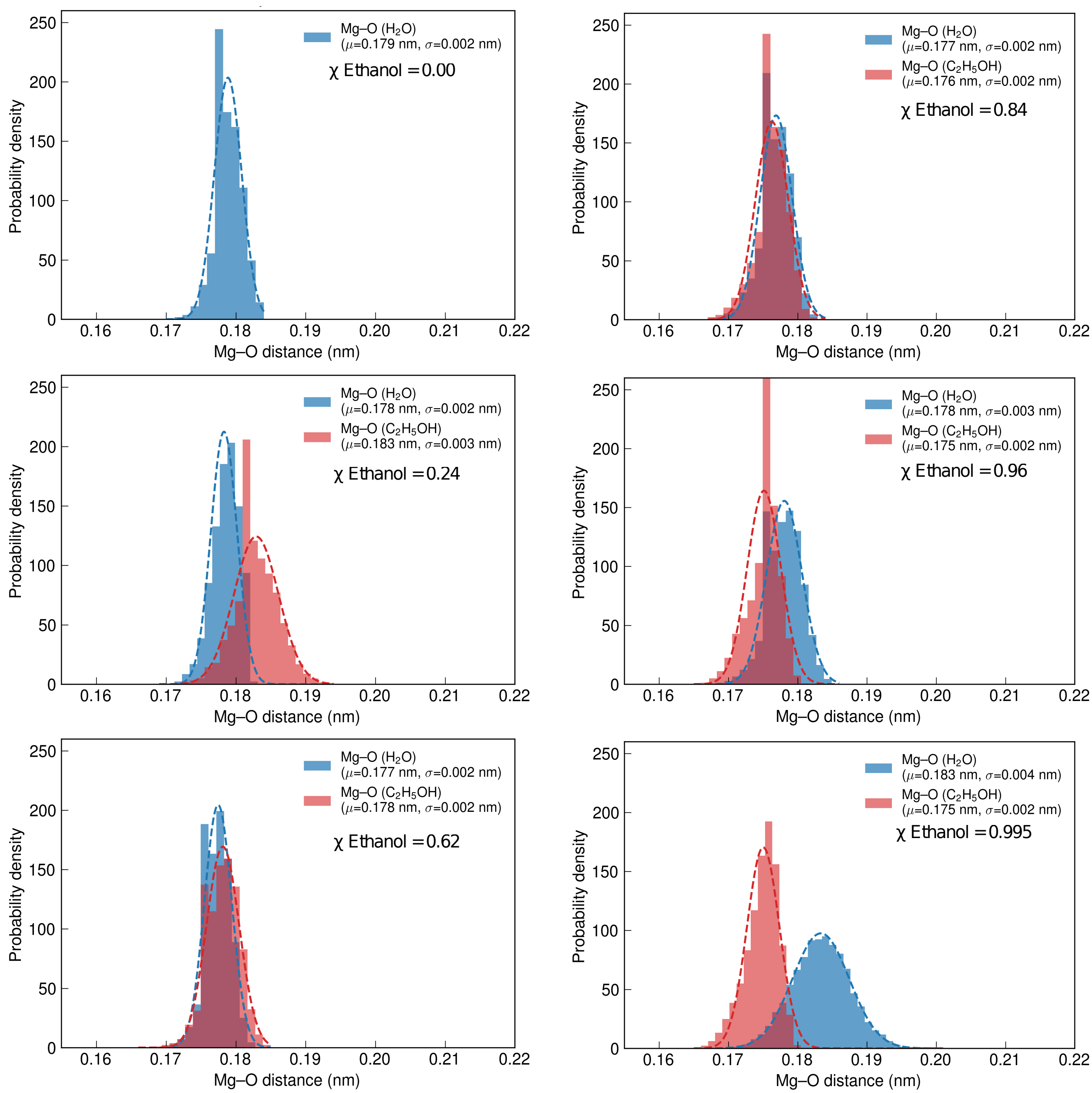

Figure S6: Probability density distribution of the minimum O–Mg^{2+} distances for water (blue) and ethanol (red) across different ethanol fractions (χ_{et}). Dotted lines represent fitted normal distributions for each system. The mean (μ) and standard deviation (σ) for the Gaussian fits are indicated in the legends. Note that water maintains a narrow MG–O distribution distance even at high ethanol concentrations.

References

(S1) *Igor Pro*, version 8.04; WaveMetrics, Inc.: Lake Oswego, OR, USA, 2019.

(S2) Kukk, E.; Snell, G.; Bozek, J. D.; Cheng, W.-T.; Berrah, N. Vibrational structure and partial rates of resonant Auger decay of the N 1s $\rightarrow 2\pi$ core excitations in nitric oxide. *Phys. Rev. A* **2001**, *63*, 062702.

(S3) Kukk, E.; Ueda, K.; Hergenhahn, U.; Liu, X.-J.; Prümper, G.; Yoshida, H.; Tamenori, Y.; Makochekanwa, C.; Tanaka, T.; Kitajima, M.; Tanaka, H. Violation of the Franck-Condon Principle due to Recoil Effects in High Energy Molecular Core-Level Photoionization. *Phys. Rev. Lett.* **2005**, *95*, 133001.

(S4) Campbell, J. L.; Papp, T. Widths of the atomic K-N7 levels. *At. Data Nucl. Data Tables* **2001**, *77*, 1–56.

(S5) Gopakumar, G.; Muchová, E.; Unger, I.; Malerz, S.; Trinter, F.; Öhrwall, G.; Lipparini, F.; Mennucci, B.; Céolin, D.; Caleman, C.; Wilkinson, I. et al. Probing aqueous ions with non-local Auger relaxation. *Phys. Chem. Chem. Phys.* **2022**, *24*, 8661–8671.

(S6) Thürmer, S.; Shinno, T.; Suzuki, T. Valence Photoelectron Spectra of Liquid Methanol and Ethanol Measured Using He II Radiation. *J. Phys. Chem. A* **2021**, *125*, 2492–2503.

(S7) Friesen, S.; Hefter, G.; Buchner, R. Cation Hydration and Ion Pairing in Aqueous Solutions of $MgCl_2$ and $CaCl_2$. *J. Phys. Chem. B* **2019**, *123*, 891–900.

(S8) Götte, L.; Parry, K. M.; Hua, W.; Verreault, D.; Allen, H. C.; Tobias, D. J. Solvent-Shared Ion Pairs at the Air–Solution Interface of Magnesium Chloride and Sulfate Solutions Revealed by Sum Frequency Spectroscopy and Molecular Dynamics Simulations. *J. Phys. Chem. A* **2017**, *121*, 6450–6459.

(S9) Boyn, J.-N.; Carter, E. A. Probing pH-Dependent Dehydration Dynamics of Mg and Ca Cations in Aqueous Solutions with Multi-Level Quantum Mechanics/Molecular Dynamics Simulations. *J. Am. Chem. Soc.* **2023**, *145*, 20462–20472.

(S10) Chatterjee, A.; Dixit, M. K.; Tembe, B. L. Solvation Structures and Dynamics of the Magnesium Chloride (Mg^{2+}–Cl^{-}) Ion Pair in Water–Ethanol Mixtures. *J. Phys. Chem. A* **2013**, *117*, 8703–8709.

(S11) Winter, B.; Weber, R.; Hertel, I. V.; Faubel, M.; Jungwirth, P.; Brown, E. C.; Bradforth, S. E. Electron Binding Energies of Aqueous Alkali and Halide Ions: EUV Photoelectron Spectroscopy of Liquid Solutions and Combined Ab Initio and Molecular Dynamics Calculations. *J. Am. Chem. Soc.* **2005**, *127*, 7203–7214.

(S12) Öhrwall, G.; Ottosson, N.; Pokapanich, W.; Legendre, S.; Svensson, S.; Björneholm, O. Charge Dependence of Solvent-Mediated Intermolecular Coster-Kronig Decay Dynamics of Aqueous Ions. *J. Phys. Chem. B.* **2010**, *114*, 17057–17061.

(S13) Muchová, E.; Gopakumar, G.; Unger, I.; Öhrwall, G.; Céolin, D.; Trinter, F.; Wilkinson, I.; Chatzigeorgiou, E.; Slavíček, P.; Hergenhahn, U.; Winter, B. et al. Attosecond formation of charge-transfer-to-solvent states of aqueous ions probed using the core-hole-clock technique. *Nat. Commun.* **2024**, *15*, 8903.

(S14) Pokapanich, W.; Bergersen, H.; Bradeanu, I. L.; Marinho, R. R. T.; Lindblad, A.; Legendre, S.; Rosso, A.; Svensson, S.; Björneholm, O.; Tchaplyguine, M.; Öhrwall, G. et al.

Auger Electron Spectroscopy as a Probe of the Solution of Aqueous Ions. *J. Am. Chem. Soc.* **2009**, *131*, 7264–7271.

(S15) Shinotsuka, H.; Da, B.; Tanuma, S.; Yoshikawa, H.; Powell, C. J.; Penn, D. R. Calculations of electron inelastic mean free paths. XI. Data for liquid water for energies from 50 eV to 30 keV. *Surf. Interface Anal.* **2017**, *49*, 238–252.

(S16) Seah, M. P.; Dench, W. A. Quantitative electron spectroscopy of surfaces: A standard data base for electron inelastic mean free paths in solids. *Surf. Interface Anal.* **1989**, *1*, 2–11.

(S17) Joung, I. S.; Cheatham III, T. E. Determination of Alkali and Halide Monovalent Ion Parameters for Use in Explicitly Solvated Biomolecular Simulations. *J. Phys. Chem. B* **2008**, *112*, 9020–9041.

(S18) Zeron, I. M.; Abascal, J. L. F.; Vega, C. A force field of Li^{+}, Na^{+}, K^{+}, Mg^{2+}, Ca^{2+}, Cl^{-}, and SO_4^{2-} in aqueous solution based on the TIP4P/2005 water model and scaled charges for the ions. *J. Chem. Phys.* **2019**, *151*, 134504.

(S19) Jorgensen, W. L.; Maxwell, D. S.; Tirado-Rives, J. Development and Testing of the OPLS All-Atom Force Field on Conformational Energetics and Properties of Organic Liquids. *J. Am. Chem. Soc.* **1996**, *118*, 11225–11236.

(S20) Martínez, L.; Andrade, R.; Birgin, E. G.; Martínez, J. M. PACKMOL: A package for building initial configurations for molecular dynamics simulations. *J. Comput. Chem.* **2009**, *30*, 2157–2164.

(S21) Caleman, C.; van Maaren, P. J.; Hong, M.; Hub, J. S.; Costa, L. T.; van der Spoel, D. Force Field Benchmark of Organic Liquids: Density, Enthalpy of Vaporization, Heat Capacities, Surface Tension, Isothermal Compressibility, Volumetric Expansion Coefficient, and Dielectric Constant. *J. Chem. Theory Comput.* **2012**, *8*, 61–74.

(S22) van der Spoel, D.; van Maaren, P. J.; Caleman, C. GROMACS molecule & liquid database. *Bioinformatics* **2012**, *28*, 752–753.

(S23) Allen, M.; Tildesley, D. *Computer Simulation of Liquids*; Clarendon Press, 1989.
